# Differing risk factors for new onset and recurrent gestational diabetes mellitus in multipara women: a cohort study

**DOI:** 10.1186/s12902-021-00920-5

**Published:** 2022-01-05

**Authors:** Li Zhang, Wei Zheng, Wenyu Huang, Lirui Zhang, Xin Liang, Guanghui Li

**Affiliations:** 1 Division of Endocrinology and Metabolism, Department of Obstetrics, Beijing Obstetrics and Gynecology Hospital, Capital Medical University, No 251, Yaojiayuan Road, Chaoyang District, Beijing, 100026 China; 2 Beijing Maternal and Child Health Care Hospital, Beijing, China; 3 Division of Endocrinology, Metabolism and Molecular Medicine, Northwestern University Feinberg School of Medicine, Chicago, USA

**Keywords:** Gestational diabetes mellitus, Recurrence, Body mass index, Risk factor, Fasting glucose

## Abstract

**Objectives:**

To assess whether recurrent gestational diabetes mellitus (GDM) and newly diagnosed GDM share similar risk factors.

**Methods:**

The study recruited a cohort of 10,151 multipara women with singleton pregnancy who delivered between 2016 and 2019 in Beijing, China. The prevalence of recurrent GDM and associated risk factors were analyzed between women with and without prior GDM history.

**Results:**

Eight hundred and seventy-five (8.6%) multipara women had a diagnosis of GDM during previous pregnancies. The prevalence of GDM and pre-gestational diabetes mellitus were 48.34% (423/875) and 7.89% (69/875) if the women were diagnosed with GDM during previous pregnancies, as compared to 16.00% (1484/9276) and 0.50% (46/9276) if the women were never diagnosed with GDM before. In women without a history of GDM, a variety of factors including older maternal age, higher pre-pregnancy body mass index (PPBMI), prolonged interval between the two pregnancies, higher early pregnancy weight gain, family history of type 2 diabetes mellitus (T2DM), maternal low birth weight, and higher early pregnancy glycemic and lipid indexes were generally associated with an increased risk of GDM at subsequent pregnancy. In women with a history of GDM, higher PPBMI, higher fasting glucose level and maternal birthweight ≥4000 g were independent risk factors for recurrent GDM.

**Conclusions:**

GDM reoccurred in nearly half of women with a history of GDM. Risk factors for recurrent GDM and newly diagnosed GDM were different. Identifying additional factors for GDM recurrence can help guide clinical management for future pregnancies to prevent GDM recurrence.

**Supplementary Information:**

The online version contains supplementary material available at 10.1186/s12902-021-00920-5.

## Introduction

Hyperglycemia first detected at any time during pregnancy that does not meet the criteria of diabetes mellitus in pregnancy is called gestational diabetes mellitus (GDM) [1, 2]. It is estimated that 21.3 million (16.2%) of women have hyperglycemia during pregnancy globally, 85.1% of which are cases of GDM [3]. GDM is associated with a number of adverse outcomes in mothers and offspring [4–6]. Women with a history of GDM have an increased risk for future glucose intolerance, including type 2 diabetes and recurrent GDM in subsequent pregnancies [7–12]. GDM also increases the risk of spontaneous abortion, fetal anomalies, macrosomia, neonatal hypoglycemia, obesity, and type 2 diabetes in the offspring [13–15].

A recent meta-analysis by Giannakou et al. showed that overweight/obese, hypothyroidism, polycystic ovary syndrome (PCOS), and family history of diabetes were convincing or highly suggestive risk factors for GDM [16]. Other reported influencing factors included maternal age, ethnicity, parity, genetic factors, lifestyles, and social economic status [17, 18]. A combination of multiple risk factors will further increase the risk of GDM. Another study by Popova et al. suggested that the combination of high BMI with high abdominal circumference and elevated fasting glucose was associated with a 13-fold increased risk of GDM as compared to women who did not have this combination of symptoms [19]. Among the reported risk factors, fasting glucose in early pregnancy has been shown to be strongly associated with adverse pregnancy outcomes [20].

By the same token, it has been consistently demonstrated that there is an increased risk of GDM in subsequent pregnancy in multipara women with a history of GDM [9, 11, 21]. A systematic review by Kim et al. reported that the risk for recurrent GDM in subsequent pregnancy was as high as 30–84% in women with prior GDM; the variations in the GDM recurrence rate were dependent on the presence of other risk factors [12]. The recurrence of GDM varied most significantly by race/ethnicity [12]. A meta-analysis by Schwartz et al. confirmed the contribution of ethnicity to GDM recurrence [22]. Additionally, other risk factors were found to be associated with recurrent GDM, including advanced maternal age, obesity, blood glucose level in the subsequent pregnancy, excessive weight gain, and short pregnancy interval [8–12, 21]. However, these factors were not consistently shown across different studies [23].

With the implementation of the diagnostic criteria for GDM by the International Association of Diabetes and Pregnancy Study Group Consensus Panel (IADPSG), the prevalence of GDM has increased significantly [24]. There have also been changes in the characteristics and prognosis of women with GDM [25]. The Chinese Medical Association also revised the diagnostic criteria in China by reference to IADPSG criteria in 2014 [26]. Yet, little is known about the prevalence and associated risk factors for recurrent GDM since the wide acceptance of the new criteria in diagnosing GDM. Furthermore, studies conducted in Asian populations are very limited.

In this study, we compared the prevalence and risk factors for GDM in subsequent pregnancy in women with or without prior GDM in a Chinese cohort. Additionally, we evaluated the effect of prior GDM and other risk factors on the risk of recurrent GDM.

## Methods

### Participants and settings

Participants were selected from an ongoing pregnancy cohort study. This cohort recruited pregnant women who intended to receive regular prenatal care and deliver at the Beijing Obstetrics and Gynecology Hospital starting in 2014. This hospital is a public hospital and the municipal maternal and child health care hospital in Beijing with an average number of births in this hospital reached 15,000 per year.

Regular prenatal care included at least 14 prenatal check-ups, including dietary guidance, measurement of anthropometric indices and blood pressure, disease screening, ultrasound monitoring of fetal growth and fetal heart monitoring, etc. All study subjects were recruited at gestational week 6–12 at their first prenatal visit to the hospital and followed every month until delivery. In this study, we selected multipara women with singleton pregnancy who entered the cohort between January 2016 and July 2019. A total of 38,003 women were screened and 10,385 multipara women was identified. Subsequently, 234 women were excluded for abortion or stillbirth, leaving 10,151 participants for analysis (Supplementary Fig. 1). The study was approved by the Ethics Committee of the Beijing Obstetrics and Gynecology Hospital (2017-KY-015-01). All procedures were performed in accordance with the Declaration of Helsinki and informed consent was obtained from all subjects.

### Experimental design

Social economic status and anthropometric data of the participants were collected at gestational week 6 to 12. Maternal birthweight ≥4000 g, pre-index pregnancy body weight and adverse pregnancy outcomes in the subsequent pregnancy were collected through a review of medical records. Pre-pregnancy body mass index (PPBMI) in kg/m^2^ was calculated as pre-pregnancy weight divided by height^2^. Adverse pregnancy history, including history of miscarriage or stillbirth, gestational hypertension, fetal macrosomia or low birth weight was also noted. Fetal macrosomia and low birth weight indicated neonatal birth weight ≥ 4000 g and < 2500 g, respectively.

Before 2012, GDM was diagnosed by using the National Diabetes Date Group two-step criteria [27]. Specifically, a screening 50 g glucose challenge test was done first. Women with 1 h plasma glucose ≥7.8 mmol/l were scheduled for a subsequent diagnostic 100 g/3 h oral glucose tolerance test (OGTT). GDM was diagnosed if two or more blood glucose values were equal to or greater than the following thresholds: fasting, 5.8 mmol/l; 1 h, 10.6 mmol/l; 2 h, 9.2 mmol/l; and 3 h, 8.1 mmol/l. Since 2012, GDM has been diagnosed by the guidelines for the management of gestational diabetes mellitus in China [26]. According to the guidelines, pregnant women with a fasting glucose level ≥ 7.0 mmol/L or random blood glucose level ≥ 11.0 mmol/L in early pregnancy are diagnosed with pre-gestational diabetes mellitus (PGDM). Women who are not diagnosed with PGDM received a 75 g OGTT at 24–28 weeks of gestation. The diagnostic criteria for GDM at 24–28 weeks of gestation is the same as the IADPSG criteria. GDM was diagnosed if one or more of the following values was met or exceeded: fasting, 5.1 mmol/L; 1 h, 10.0 mmol/L; and 2 h, 8.5 mmol/L. [28]. Women with a GDM history in any of their previous pregnancies were referred for GDM diagnosis by the above criteria. Women in subsequent pregnancy were all diagnosed by the IADPSG criteria.

Additionally, venous blood samples were collected following an overnight fast at 7–13 weeks (early gestation) and 24–28 weeks of gestation. Total cholesterol and triglycerides were determined using enzymatic methods, while high density lipoprotein-cholesterol and low density lipoprotein-cholesterol were directly measured (Architect ci8200, Abbott Laboratories, USA). Blood glucose level was measured using the glucose oxidase method.

### Statistics

All statistical analysis was conducted using SAS version 9.3 (SAS Institute Inc., Cary, NC, USA). Characteristics of the participants were reported as mean ± standard deviation for continuous variables and as numbers and percentages for categorical variables. Comparison of the characteristics between women with and without GDM history was made by Student’s t-test or the chi-squared test. The frequency of PGDM and GDM in the subsequent pregnancy was compared between women with prior GDM and women without GDM history. Risk factors were analyzed for recurrent GDM and new onset GDM in the subsequent pregnancy by multivariate logistic regression models. The potential risk factors included maternal age, gravidity, parity, interval between the two pregnancies, adverse pregnancy history, family history of T2DM, PCOS, inappropriate maternal birthweight, PPBMI, early pregnancy weight gain, fasting glucose, total cholesterol, triglycerides, high density lipoprotein-cholesterol, and low density lipoprotein-cholesterol. Final models were determined by stepwise forward variable selection procedure and covariates significant at 5% were included in the model. Further analysis was conducted to calculate ORs for the combination of the risk factors of recurrent GDM.

## Results

Among 10,151 multipara women, 876 (8.6%) subjects had a history of GDM. The prevalence of newly developed PGDM and GDM in the subsequent pregnancy was higher in women with prior GDM than women without a history of GDM (7.89% vs. 0.50%, *p* < 0.0001 and 48.34% vs. 16.00%, *p* < 0.0001).

We further compared the characteristic of women with and without a history of GDM by excluding PGDM (*n* = 10,036) (Table 1). Women with previous GDM showed advanced age, higher proportion of adverse pregnancy history, family history of T2DM, PCOS, maternal low birth weight, hypertensive disorders complicating pregnancy and higher PPBMI. Additionally, higher fasting glucose level and lipid levels were also observed in women with prior GDM than women without a history of GDM. Women with a history of GDM also had higher fasting, 1 h and 2 h glucose levels at 24–28 weeks of gestation in the subsequent pregnancy than women without GDM history.

**Table 1:** Characteristics and early pregnancy metabolic factors of participants with and without GDM history by excluding PGDM

	GDM history	No GDM history	*p*-value^†^
Basic characteristics			
N	806	9230	
Age, year, mean ± SD	35.43 ± 3.33	35.08 ± 3.57	0.007
Gravidity			0.4
2nd, n(%)	376 (46.65)	4443 (48.14)	
3rd or more, n(%)	430 (53.35)	4787 (51.86)	
Parity			0.1
2nd, n(%)	770 (95.53)	8912 (96.55)	
3rd or more, n(%)	36 (4.47)	318 (3.45)	
Interval between the two pregnancies, mean ± SD	5.03 ± 2.60	6.50 ± 3.43	< 0.0001
Adverse pregnancy history			
History of gestational hypertension, n(%)	51 (6.33)	283 (3.07)	< 0.0001
History of fetal macrosomia, n(%)	76 (9.43)	602 (6.52)	0.002
History of fetal low birth weight, n(%)	3 (0.37)	39 (0.42)	0.8
Family history of T2DM, n(%)	229 (28.41)	1240 (13.43)	< 0.0001
PCOS, n(%)	44 (5.46)	228 (2.47)	< 0.0001
Maternal birthweight			< 0.0001
< 2500 g, n(%)	52 (6.45)	317 (3.43)	
2500 g ~ 3999 g, n(%)	697 (86.48)	8050 (87.22)	
≥ 4000 g, n(%)	57 (7.07)	863 (9.35)	
Height, cm, mean ± SD	162.0 ± 4.9	162.5 ± 4.8	0.02
Pre-pregnancy weight, kg, mean ± SD	60.3 ± 9.9	58.6 ± 9.0	< 0.0001
PPBMI, kg/m^2^, mean ± SD	22.9 ± 3.4	22.2 ± 3.2	< 0.0001
Early pregnancy weight gain, kg, mean ± SD	1.0 ± 1.7	1.0 ± 1.8	0.8
Gestational age of weight gain measured, week, mean ± SD	8.1 ± 2.1	8.4 ± 2.4	0.007
hypertensive disorders complicating pregnancy, n(%)	58 (7.20)	577 (6.25)	0.3
Glycolipid index			
Early pregnancy metabolic factors			
FBG, mmol/L, mean ± SD	4.89 ± 0.44	4.70 ± 0.36	< 0.0001
Total cholesterol, mmol/L, mean ± SD	4.30 ± 0.72	4.26 ± 0.73	0.2
Triglycerides, mmol/L, mean ± SD	1.33 ± 0.60	1.19 ± 0.55	< 0.0001
High density lipoprotein-cholesterol, mmol/L, mean ± SD	1.50 ± 0.32	1.57 ± 0.33	< 0.0001
Low density lipoprotein-cholesterol, mmol/L, mean ± SD	2.24 ± 0.58	2.17 ± 0.58	0.0002
GDM diagnosed during 24 ~ 28 weeks of gestation, n(%)	423 (48.34)	1484 (16.00)	< 0.0001
OGTT values in previous normoglycemic participants during 24 ~ 28 weeks of gestation			
Fasting blood glucose level, mmol/L	4.80 ± 0.52	4.50 ± 0.41	< 0.0001
1 h blood glucose level, mmol/L	9.07 ± 1.74	7.59 ± 1.61	< 0.0001
2 h blood glucose level, mmol/L	7.54 ± 1.60	6.56 ± 1.29	< 0.0001

*GDM* gestational diabetes mellitus, *T2DM* type 2 diabetes mellitus, *PCOS* polycystic ovary syndrome, *PPBMI* pre-pregnancy body mass index, *FBG* fasting blood glucose, *PGDM* pre-gestational diabetes mellitus, *OGTT* oral glucose tolerance test

^†^p-value was calculated by student t-test or chi-square test

Risk factors for GDM in the subsequent pregnancy differed significantly between women with a history of GDM and those without prior GDM (Table 2). In women without a history of GDM, a variety of factors including advanced maternal age, higher PPBMI, prolonged interval between the two pregnancies, higher early pregnancy weight gain, family history of T2DM, maternal low birth weight and higher early pregnancy glycemic and lipid indexes were generally associated with an increased risk of GDM at subsequent pregnancy. In contrast, in women with a prior diagnosis of GDM, only a few factors including PPBMI, higher fasting glucose level in early pregnancy and maternal birthweight ≥4000 g were identified to be strongly related to GDM recurrence.

**Table 2:** Risk factors for recurrent GDM and new developed GDM in multipara

	Recurrent GDM OR^a^ (95% CI)	New developed GDM OR^a^(95% CI)
Age^b^	–	1.06 (1.04 ~ 1.09)
PPBMI^b^	1.07 (1.02 ~ 1.12)	1.09 (1.07 ~ 1.11)
Interval between the two pregnancies	–	1.05 (1.03 ~ 1.07)
Early pregnancy weight gain^b^	–	1.08 (1.05 ~ 1.12)
Family history of T2DM	–	1.52 (1.30 ~ 1.78)
Maternal birthweight	–	–
< 2500 g	1.32 (0.72–2.40)	1.47 (1.10 ~ 1.96)
2500 g ~ 3999 g	1	1
≥ 4000 g	1.84 (1.02–3.31)	0.87 (0.70 ~ 1.08)
FBG^b^	2.26 (1.58 ~ 3.25)	4.58 (3.88 ~ 5.41)
Triglyceride^b^	–	1.71 (1.54 ~ 1.90)

*GDM* gestational diabetes mellitus, *PPBMI* pre-pregnancy body mass index, *T2DM* type 2 diabetes mellitus, *FBG* fasting blood glucose

^a^OR was adjusted for other variables in this table by multivariate regression model. Variables included in the models were selected by stepwise forward variable selection procedure

^b^Age (y), PPBMI (kg/m^2^), early pregnancy weight gain (kg), FBG (mmol/L), and triglyceride (mmol/L) were included in the model as continuous variables. ORs were calculated for each unit increase of the factors

Table 3 demonstrates the accumulative risk of recurrent GDM based on the number of risk factors present. One more risk factor in addition to a history of GDM increased the absolute risk of GDM recurrence from 44.44 to 58.02% with an OR of 1.73 (95% CI = 1.27–2.35), while the existence of two and more risk factors in addition to a history of GDM resulted in a more than 1.5-fold increase in the risk of recurrent GDM from 44.54 to 78.82% with an OR of 4.65 (95% CI = 2.68–8.08). The prevalence of recurrent GDM was contributed most by PPBMI and higher fasting glucose in early pregnancy (FBG ≥5.15 mmol/l), followed by maternal birth weight ≥ 4000 g.

**Table 3:** Combined effect of risk factors on prevalence of recurrent GDM

	Recurrent GDM, n(%)	OR(95% CI)
GDM history	204 (44.44)	1
GDM history + one risk factor^a^	152 (58.02)	1.73 (1.27–2.35)
GDM history + two risk factor or more^a^	67 (78.82)	4.65 (2.68–8.08)
GDM history + FBG ≥ 5.15 mmol/L in early pregnancy (≥75%)	133 (65.84)	2.09 (1.50–2.91)
GDM history + PPBMI≥25 kg/m^2^	121 (67.22)	2.20 (1.55–3.12)
GDM history + Maternal birthweight≥4000 g	37 (64.91)	1.74 (0.99–3.05)

*GDM* gestational diabetes, *FBG* fasting blood glucose, *PPBMI* pre-pregnancy BMI

^a^Risk factors included were higher FBG in early pregnancy (≥75%), PPBMI≥25 kg/m^2^, and Maternal birthweight≥4000 g

## Discussion

In this cohort study, we found that the risk of GDM in subsequent pregnancy in women with a history GDM was three times as high as that in women without history of GDM. Furthermore, there was significant difference in the risk factors for newly diagnosed GDM and recurrent GDM.

Our study confirmed previous observations that women with a history of GDM are at increased risk for recurrent GDM in a subsequent pregnancy. A meta-analysis by Schwartz et al. found a pooled GDM recurrence rate of 48%, which is similar to our finding [22]. It has been observed that the recurrence rate of GDM is highly variable across different ethnic groups. Previously reported GDM recurrence rates have varied widely from 30 to 84%, depending on the ethnicity of the study subjects [12, 21, 29]. Studies on GDM recurrence in Asian populations are limited. A recent study in Korea women found that GDM recurred in approximately half of women with prior GDM [11]. Few studies are available regarding GDM recurrence in Chinese women, largely due to the long-term one-child policy in China. A study by Wang et al. reported that the frequency of recurrent GDM was 55% [30], which was approximately the sum of the prevalence of recurrent GDM and PGDM in our study. Since the abolition of the one-child policy in 2016 [31], there has been a significant increase in children born to mothers at an advanced age, drawing the attention to elevated incidence of gestational complications.

In our cohort, the risk factors associated with newly diagnosed GDM were significantly different from those associated with recurrent GDM. In multipara women without prior GDM, various risk factors were identified to be associated with GDM in the subsequent pregnancy, including advanced maternal age, higher PPBMI, increased interval between the two pregnancies, family history of T2DM, maternal low birth weight and higher early pregnancy metabolic factors such as blood glucose level and blood lipid levels. These identified risk factors for GDM are similar to those shown in previous studies of new onset GDM [32, 33]. In contrast, recurrent GDM was associated with relatively few risk factors.

It is notable that several seemingly important risk factors were identified only in the new onset GDM group, but not in the recurrent GDM group, including advanced maternal age, family history of T2DM, excessive gestational weight gain in early pregnancy and increased lipid profile. One plausible cause for this observed difference is that a GDM history *pe* se in women with recurrent GDM may represent an integrative outcome of a dysregulated lipid metabolism and other risk factors, which may have already encompassed these risk factors.

Nevertheless, a few distinct risk factors including higher PPBMI, higher fasting glucose level in early pregnancy and maternal birthweight ≥4000 g have conferred additional risk for recurrent GDM. Maternal obesity has been shown to be associated with recurrent GDM in previous studies [11, 21, 22, 29], which was consistent with our findings. One plausible explanation is that the inflammatory milieu plays an important role in development of GDM in obese women [17]. Chronic low grade inflammation is a potential link between obesity and insulin resistance. Chemokines in adipose tissue induce the infiltration of monocytes and macrophages, which promote the secretion of pro-inflammatory factors and reduce adiponectin levels, resulting in insulin resistance [34]. Excessive peripheral insulin resistance decreases glucose uptake by skeletal muscle and adipose tissue, leading to glucose intolerance [17].

A higher fasting glucose level has been considered as an important factor for GDM prediction [35, 36], which is consistent with the results of this study. It has been shown that women who develop GDM during pregnancy have metabolic dysfunction before conception, such as pancreatic β-cell defects and increased insulin resistance. They maintain normoglycemia in early pregnancy because of the compensated adaptation of pancreatic β-cells. However, as physiological insulin resistance increases during pregnancy, the insulin response becomes inadequate [17]. Previous studies have indicated that a higher glucose level in the previous pregnancy is a risk factor for recurrent GDM [30]. However, a majority of pregnant women could not recall their specific glycemic values in the previous pregnancy. Testing the fasting glucose level in early pregnancy in the subsequent pregnancy is more feasible and valuable to predict recurrent GDM, and thereby has more practical significance for the clinical management of high risk groups.

The uniqueness of the current study is that we identified drastically distinct profile of risk factors between new onset GDM and recurrent GDM. Although a GDM history *pe se* is one of the most important indices predicting GDM recurrence, identifying additional risk factors helps us develop more individualized intervention strategies, such as weight and glucose management between pregnancies. There are certain limitations to our study, including the retrospective nature of data collection in the previous pregnancy and the collection of maternal birth weight. Recall bias may blur the effect of glucose status in previous pregnancy on recurrence of GDM in the subsequent pregnancy. Furthermore, different diagnostic criteria for GDM were implemented before and after 2012 in this study. Consequently, women who give birth before 2012 and have slightly elevated blood glucose levels according to the new criteria were excluded from this study. Therefore, the characteristic of the participants changed temporally, which may confound the association between the risk factors and GDM. Nevertheless, a uniform criteria was used to diagnose GDM in the subsequent pregnancy. Additionally, we could not evaluate the effect of ethnicity since the study population was of a single ethnicity.

In summary, this study revealed that GDM recurred in approximately half of women with a history of GDM. Higher fasting glucose level, high maternal birthweight and maternal overweight and obesity confer additional risk for recurrent GDM in addition to a history of GDM. Up to 77.42% of women with prior GDM had recurrent GDM if these additional risk factors were present. Recognizing these additional factors antepartum can help to identify a high risk population in order to prevent recurrent GDM, and may also provide an opportunity to explore pathogenesis of recurrent GDM.

## Supplementary Information

**Additional file 1.**

## Data Availability

Dataset used in this study is available upon reasonable request to the corresponding author via liguanghui@ccmu.edu.cn
