# Supplementary material for: Differing risk factors for new onset and recurrent gestational diabetes mellitus in multipara women: a cohort study
Source: BMC Endocr Disord. 2022 Jan 5;22:3. doi: 10.1186/s12902-021-00920-5 (PMC8728925; doi:10.1186/s12902-021-00920-5)
Supplement: Supplementary file 1 — Additional file 1. [file 12902_2021_920_MOESM1_ESM.pptx]

## Slide 1
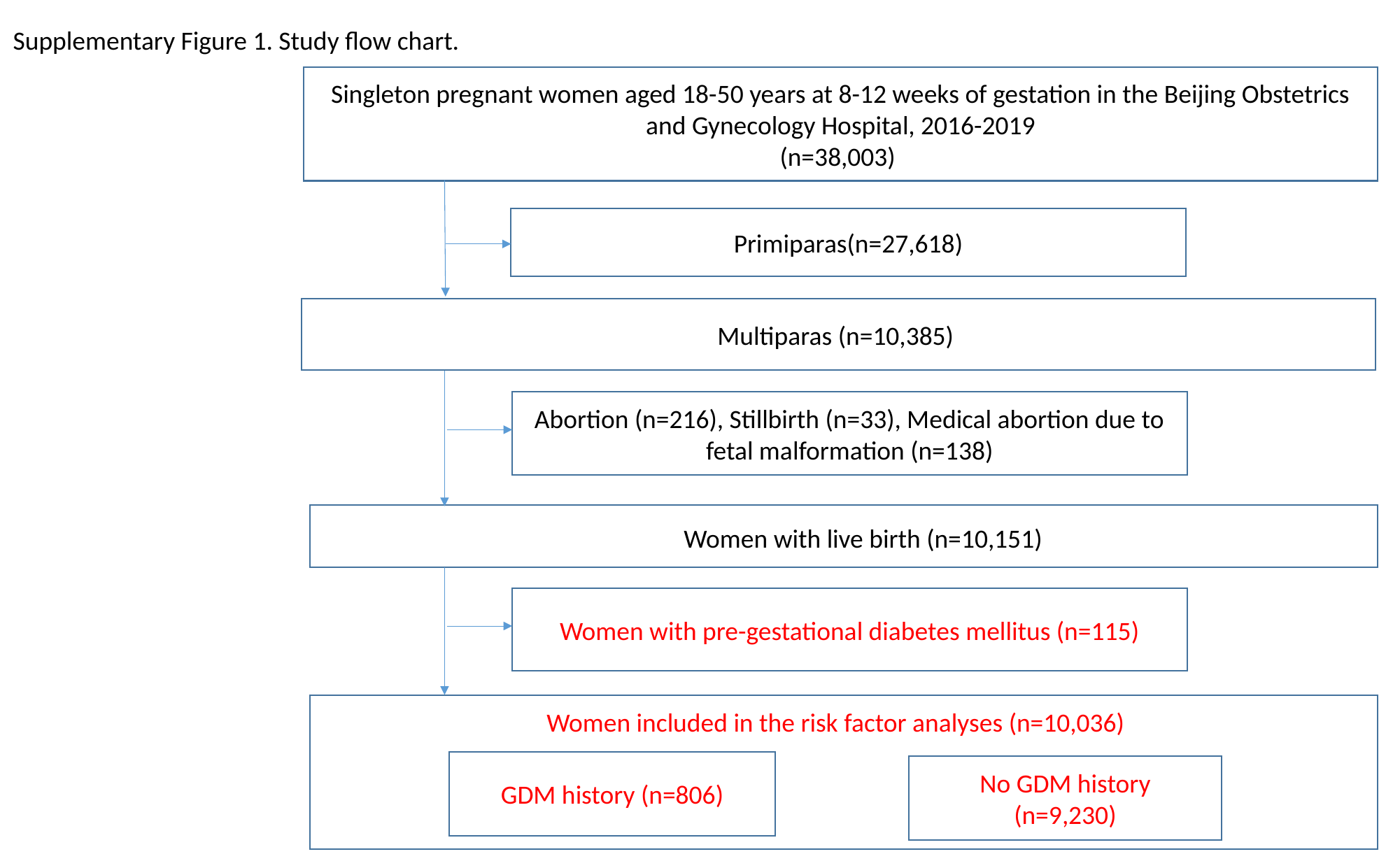

Supplementary Figure 1. Study flow chart.
Singleton pregnant women aged 18-50 years at 8-12 weeks of gestation in the Beijing Obstetrics and Gynecology Hospital, 2016-2019
(n=38,003)
Primiparas(n=27,618)
Multiparas (n=10,385)
Abortion (n=216), Stillbirth (n=33), Medical abortion due to fetal malformation (n=138)
Women with live birth (n=10,151)
Women with pre-gestational diabetes mellitus (n=115)
Women included in the risk factor analyses (n=10,036)
GDM history (n=806)
No GDM history
(n=9,230)
